# Control of Airway Tube Diameter and Integrity by Secreted Chitin-Binding Proteins in *Drosophila*

**DOI:** 10.1371/journal.pone.0067415

**Published:** 2013-06-24

**Authors:** Katarína Tiklová, Vasilios Tsarouhas, Christos Samakovlis

**Affiliations:** Department of Molecular Biosciences, The Wenner-Gren Institute, Stockholm University, Stockholm, Sweden; Alexander Flemming Biomedical Sciences Research Center, Greece

## Abstract

The transporting function of many branched tubular networks like our lungs and circulatory system depend on the sizes and shapes of their branches. Understanding the mechanisms of tube size control during organ development may offer new insights into a variety of human pathologies associated with stenoses or cystic dilations in tubular organs. Here, we present the first secreted luminal proteins involved in tube diametric expansion in the *Drosophila* airways. *obst-A* and *gasp* are conserved among insect species and encode secreted proteins with chitin binding domains. We show that the widely used tracheal marker 2A12, recognizes the Gasp protein. Analysis of *obst-A* and *gasp* single mutants and *obst-A; gasp* double mutant shows that both genes are primarily required for airway tube dilation. Similarly, Obst-A and Gasp control epidermal cuticle integrity and larval growth. The assembly of the apical chitinous matrix of the airway tubes is defective in *gasp* and *obst-A* mutants. The defects become exaggerated in double mutants indicating that the genes have partially redundant functions in chitin structure modification. The phenotypes in luminal chitin assembly in the airway tubes are accompanied by a corresponding reduction in tube diameter in the mutants. Conversely, overexpression of Obst-A and Gasp causes irregular tube expansion and interferes with tube maturation. Our results suggest that the luminal levels of matrix binding proteins determine the extent of diametric growth. We propose that Obst-A and Gasp organize luminal matrix assembly, which in turn controls the apical shapes of adjacent cells during tube diameter expansion.

## Introduction

Tube length and diameter are major determinants of flow rates in tubular organs. The generation of appropriate tube dimensions during organ morphogenesis is critical for tissue function and animal homeostasis. Consequently an important challenge for research aiming to understand tubular organ development is to elucidate the acquisition of stereotyped dimensions and proportions in the branches of a tubular network. The major airways of the *Drosophila* tracheal network consist of a single epithelial cell layer and provide a simple system for the genetic dissection of tube size control. Tracheal tube expansion occurs without increases in cell numbers and experimental alterations in cell numbers do not affect tracheal tube dimensions. These results suggested that tube growth in the embryonic and larval trachea relies on cell rearrangements and cell shape adjustments to form tubes of specified sizes. The analysis of many mutants with selective tracheal tube overgrowth defects has elucidated some of the cellular mechanisms in epithelial tube size regulation [1]–[7].

A central role in tracheal tube size regulation has been ascribed to the structure and dynamic modifications of the apical extracellular matrix. Diametric tube expansion is preceded by the generation of a transient luminal cable composed of chitin fibrils. Chitin and associated proteins also assemble into a complex apical matrix, the taenidia, which is tightly juxtaposed with the apical surface of the epithelium. Whereas the luminal chitin cable is cleared from the tube before larval hatching to enable gas filing, the taenidial matrix remains and is thought to reinforce the larval network [8], [9]. Mutations in genes involved in chitin biogenesis and assembly result in irregular diametric expansion leading to locally constricted and dilated tubes in the mutants [9]–[11]. In addition, these mutants show overelongated tracheal branches at the end of embryogenesis. This led to the hypothesis that the expanding luminal chitin filament coordinates the epithelial cell shape rearrangements during tube growth. The importance of luminal chitin in tracheal tube size control is further highlighted by the analysis of *vermiform* (*verm*) and *serpentine* (*serp*) mutants, which show luminal matrix defects and tube overelongation. *verm* and *serp* encode luminal putative chitin deacetylases, suggesting that the acetylated chitin matrix restricts cell extension and tube elongation [12], [13]. Tube overelongation is also the hallmark phenotype for another group of mutants including genes involved in the assembly of septate epithelial junctions (SJs). Insect SJS are functionally similar to the vertebrate tight junctions controlling the paracellular traffic between epithelial cells. “SJ mutants” show impaired Verm and Serp secretion into the lumen indicating that a function of SJ proteins may be to facilitate the apical targeting of chitin modifying enzymes. An additional mechanism by which SJ proteins control tube size is, through the regulation of the subcellular localization of apical polarity proteins like Crumbs and aPKC [4], [14]. Recently, the non-receptor tyrosine kinase Src42A has been identified as a driver of tube elongation. *src42A* mutants show short tubes and Src42A overexpression can cause overelongation. It is suggested that Src42A activation promotes the axial extension of the tracheal cells along the longitudinal axis of the tubes to promote elongation [15], [16]. Collectively, the results from several studies indicate that Src42A activation promotes elongation by controlling the apical cytoskeleton and cell junctions whereas the dynamic modifications of the apical ECM and suppression of Crb targeting to the apical membrane control the termination of tube elongation.

Despite the progress in identifying mechanisms of tube elongation, we know little about tracheal tube growth in the radial axis. A burst of epithelial secretory activity deposits luminal proteins in the tubes and precedes diametric expansion. Mutations affecting genes involved in the transport steps of secreted proteins from and to the endoplasmatic reticulum (ER) like *sar1*, show a strong reduction in tube diameter and defective luminal matrix [17]–[21]. There are several models attempting to explain the role of secretion in diametric tube expansion. One model postulates that the secretion burst of luminal proteins underlies the modification of the chitininous ECM. Unknown secreted proteins are thought to modify the biophysical properties of luminal chitin and cause it to swell to inflate the tube. Alternatively, the secretory burst may deliver the necessary apical membrane from the ER to the apical cell surface to meet the needs of tube diameter expansion. Finally, apical secretion may deliver regulators of osmotic pressure to the apical membrane and thereby generate a force for tube inflation. The later model involving the apical delivery of a tube-wide osmotic dilator seems unlikely since individual cells expressing a wild-type copy of Sec24 in *sec24* mutants can support a local tube dilation in an otherwise stenotic tracheal tube [16]. Thus, although the importance of developmentally regulated apical secretion is at the heart of diametric tube expansion in the trachea, neither the secretory cargoes nor the cellular mechanisms underlying the secretion mediated tube expansion are known.

The aim of this work was to identify and characterize secreted proteins involved in diametric tube expansion. We present the analysis of *obst-A* and *gasp* mutants and show that both genes are required for larval growth and diametric tracheal tube expansion. They encode secreted luminal proteins with chitin binding domains and are required for intraluminal chitin filament assembly and taenidial integrity. We identify the Gasp protein as the unknown antigen recognized by the widely used antibody marker 2A12. The deletion of *obst-A* or *gasp* genes shows mildly stenotic tubes but the diametric expansion phenotype becomes exaggerated in the double mutants. Conversely, concurrent overexpression of both proteins in wild-type animals leads to localized tube dilations. Our analysis argues that Obst-A and Gasp modify the taenidial matrix to provide an expanding mold that determines airway tube expansion.

## Materials and Methods

### Drosophila Strains

The *obst-A* null mutant was generated by flip FRT technique using two piggyBac elements (f02348, f03687). w^1118^ was used as the wild-type strain. *gasp* (P{XP}d02290) mutant is described in Flybase. Crosses for ectopic expression using *btl*-GAL4, *en*-GAL4 and *69B*-GAL4 drivers were performed at 25°C. In all experiments CyO, TM3 and FM7c balancer strains carrying *GFP* or *lacZ* transgenes were used as necessary to identify embryos with the desired genotypes.

### Immunostaining and Transmission Electron Microscopy (TEM)

Immunolabeling and TEM were performed as described [13], [22]. The following antibodies were used: mouse anti-tracheal luminal 2A12 (DSHB), rat anti-DE-Cad (DSHB), mouse anti-Crb (DSHB), mouse anti-αSpectrin (DSHB), rabbit anti-GFP (Molecular Probes), gp anti-Verm [13], gp anti-MTf [23], gp anti-Gasp [17], rabbit anti-Uif [24], rabbit anti-β-gal (Cappel), fluorescein-conjugated ChtB (New England BioLabs). Images were acquired with a Zeiss LSM 510-META confocal and Tecnai G2 Spirit BioTWIN electron microscope. Images were post-processed with Adobe Photoshop.

### Molecular Biology and Western Blotting

The UAS-*obst-A* transgene was generated by subcloning the insert of LD43683 into pUAST. The constructs used for protein expression in BL21 cells were the *Melanotransferrin* (*MTf)* cDNA fused to the 6×HisTag [23] and the *gasp* cDNA fused to 6×HisTag [17]. The pET28a plasmid vector was used as a negative control. Expressed proteins were analyzed by immunoblotting in a standard western blotting procedure.

### Live Imaging

Embryos were dechorionated in 3% sodium hypo-chloride for 2–3 min, mounted in to a slide with a gas permeable membrane [17] and imaged with a CCD camera (AxioCam MRm) attached to an AxioImagerZ1 (Zeiss) microscope by using a 20x/0.75NA Plan-APOCHROMAT objective (Zeiss). Time-lapse movies were created from Z-stack projections using NIH ImageJ (http://rsb.info.nih.gov/ij/).

### Morphometric Analysis and Statistics

To asses embryonic tracheal tube size (tube diameter or length), wild-type, *obst-A*, *gasp* and *obst-A; gasp* mutant embryos were stained with the apical markers Crumbs (Crb) or Uninflatable (Uif) and imaged by confocal microscopy (LSM510, Zeiss). Confocal digital micrographs (Maximum intensity projections) of tracheal metameres 4–6 were used to estimate diameter or length. Tube diameter in each embryo was calculated as the average of three different measurements within each metamere. Tube length was measured by tracing the apical localization of the Crb or Uif immunostainings. We used at least 6 embryos at stage 16.1 for the diameter and length measurements of each genotype. Larval body size measurements were done in anesthetized living larvae, imaged by wide field microscopy (Axioplan2, Zeiss). Measurements were conducted using the AxioVision 4.8.2 (Zeiss) software. Results are presented in bar graphs as the means ± standard deviation unless otherwise indicated. In all cases a two-tailed distribution unpaired Student’s *t*-test was used to estimate the statistical significance of the differences between wild-type and each mutant.

## Results

### Obst-A and Gasp are Expressed and Secreted into the Airways during Tube Expansion

**Figure 1:**
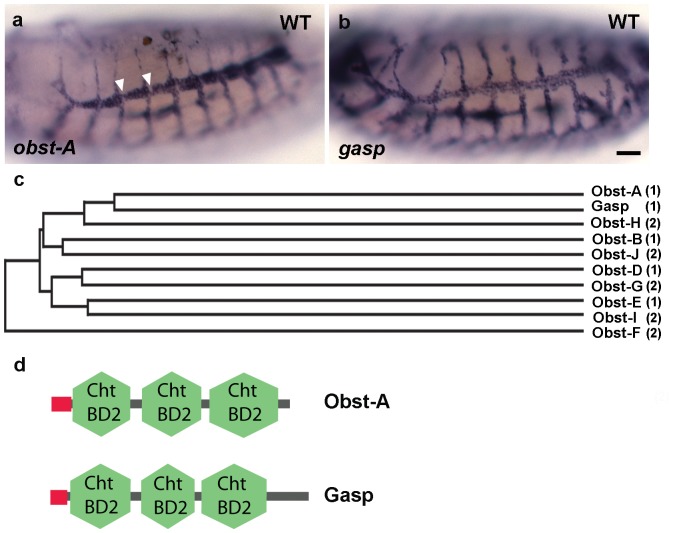
Obst-A and Gasp and their expression. (a, b) Whole-mount in situ hybridization of wild-type embryos with *obst-A* (a) and *gasp* (b) probes. *obst-A* and *gasp* mRNA are detected in tracheal cells from stage 13 onward. Note the gaps in *obst-A* expression correspond to the position of the fusion cells (arrowheads). Scale bar: 25 µm (c) A phylogenetic tree of Obstructor proteins based on ClustalW method. The number in brackets indicates, which subgroup of the Obstructor family (1 or 2) each gene belongs to. (d) Schematic representation of the Obst-A and Gasp protein domains. N-terminal signal sequence is followed by three chitin-binding domains type 2 (CBD2).

We surveyed the BDGP gene expression database to identify genes that are expressed in the trachea and encode putative secreted proteins [25]. Several members of the Obstructor gene family were strongly expressed in the airways during the tube expansion period. This gene family is conserved among insects and can be divided into two subgroups, based on protein domain arrangement [26]. We focused on the Obstructor subgroup 1 because it contains genes with distinct tracheal expression patterns. *obst-A, B, C* and *D* are weakly expressed during early embryogenesis but their expression increases later, accompanying the initiation of cuticle formation in ectodermal tissues and diametric tube expansion in the airways [26]. *obst-A* (Fig. 1a) and *obst-B* are both expressed in the trachea and epidermis; *gasp* (*obst-C*) (Fig. 1b) shows expression in the tracheal system, foregut and salivary glands. Interestingly, *gasp* mRNA levels were homogeneous in all cells of the tracheal network, whereas *obst-A* mRNA was reduced or absent in cells located at regular intervals along the DT airways. The remaining of the tracheal cells expressed *obst-A* at high levels, suggesting that *obst-A* expression is differentially regulated in the DT cells. *obst-D* and *obst-E* are mostly detected in the midgut and epidermis. The expression of the subgroup 2 genes is not elevated during embryogenesis, arguing against a role of them in tube morphogenesis [26]. To examine the phylogenetic relationships among the *obst* family members, we constructed a phylogenetic tree based on the predicted protein sequences (Fig. 1c). *obst-A* and *gasp* are the most closely related genes expressed in the trachea. Alignment of the protein sequences revealed 37% identity. The predicted proteins contain a signal peptide at the amino terminus, followed by three chitin-binding type 2 domains (CBD2) (Fig. 1d). CBD2 is an extracellular domain that contains six conserved cysteines that probably form three disulphide bridges. These domains are connected with linker regions of similar length in both proteins. The features of the two proteins and the timing of their expression before the tube expansion prompted to explore Obst-A and Gasp function in the tracheal tubes.

**Figure 2:**
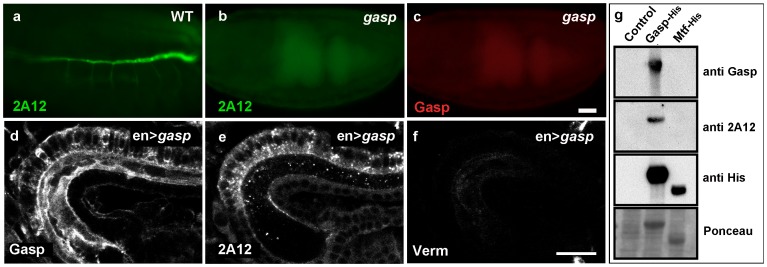
Gasp is the unknown antigen recognized by mAb 2A12. (a) Wild-type embryo stained with antibodies against the luminal antigen 2A12. (b, c) *gasp* mutant embryo stained with 2A12 (b) and Gasp (c) antibodies. 2A12 and Gasp staining is not detected in *gasp* mutant. Scale bar: 25 µm (d–f) Confocal sections of embryonic hindgut of *en>gasp* embryos labeled with Gasp (d), 2A12 (e) and Verm (f) antibodies. The luminal staining is more pronounced with the Gasp antiserum. Gasp protein expressed in the dorsal part of the hindgut is recognized by Gasp and 2A12 antibodies, but not by the Verm antibody. Scale bar: 10 µm. (g) Western blots of bacterial extracts expressing recombinant Gasp-His and MTf-His. Gasp-His protein is detected by the anti Gasp, mAb2A12 and anti-His antibodies, MTf-His protein is only detected by the anti-His antibody. Expression of both proteins is visualized by Ponceau staining. Empty vector was used as a negative control.

We first generated a null *obst-A^Δ1^* mutant (referred to as *obst-A* mutant in the remaining of the text) by the FLP-FRT method [27]. mRNA *in situ* hybridizations of mutant and wild-type embryos confirmed that *obst-A* was deleted since we could not detect any *obst-A* mRNA expression in the *obst-A* mutants (Fig. S1b). *obst-A* mutants showed larval lethality, which could be rescued by the expression of transgenic *obst-A* in the mutants using the ectodermal driver *69BGal4* indicating that the deletion does not affect any neighboring genes. For *gasp*, we used a transposon-induced mutation, which carries a P-element 66 bp upstream of the predicted translation initiation site of the *gasp* gene and is larval lethal. We analyzed *gasp* mutants using a polyclonal antiserum generated against recombinant Gasp [17]. We could not detect any Gasp protein in the *gasp* mutants suggesting that transposon insertion abolishes *gasp* expression in homozygous embryos (Fig. S1d). During these experiments we also noticed that the Gasp protein and the unknown antigen recognized by the 2A12 antibody show a similar pattern of protein localization in the trachea. Initially during embryonic stages 13 and 14, both Gasp and the 2A12 antigen are predominantly cytoplasmic. At stage 15 they are both deposited into the lumen and by stage 16, we could only detect a luminal signal for both antigens. Surprisingly, when we stained *gasp* mutant embryos for both Gasp and the 2A12 antigen we did not detect any signal from either of these antibodies (Fig. 2b, c). Parallel staining for other luminal markers in the mutants did not reveal any notable changes in their localization (Fig 4c, g). These results implied that the antigen for 2A12 is either encoded by *gasp* or by another gene, whose expression is severely reduced in *gasp* mutants. To test this, we first expressed Gasp protein using the *en*-GAL4 driver in the dorsal part of the hindgut, where *gasp* is not normally expressed. We stained *en>gasp* embryos for Gasp (Fig. 2d), 2A12 (Fig. 2e) and Verm an unrelated chitin-binding protein (Fig. 2f). Ectopic Gasp was detected in the cells of the dorsal hindgut and in the hindgut lumen by both the polyclonal Gasp antiserum and by the 2A12 monoclonal antibody, but not by the Verm antiserum. This suggested that the epitope recognized by the 2A12 antibody is part of the *gasp* open reading frame. The Gasp antiserum produced a stronger signal in the hindgut lumen compared to 2A12 indicating that the guinea pig antiserum more readily recognizes additional epitopes on Gasp (Fig. 2d, e).

We further explored whether the 2A12 mAb recognizes recombinant Gasp protein expressed in bacteria. We prepared extracts from cultures expressing either a His-tagged version of Gasp, an unrelated His-tagged MTf protein [23] and a vector expressing only the tag as a control (Fig. 2g). We probed Western blots of the extracts with the Gasp antiserum, the 2A12 mAb and an antiserum against the His-tag. All three reagents detected the Gasp-His tagged protein, whereas MTf-His was only recognized by the anti-His-tag antibody. This indicates that recombinant Gasp is the target of the 2A12 mAb and identifies the unknown antigen recognized by a widely used airway marker in *Drosophila*.

### Obst-A and Gasp Control Cuticle Morphogenesis and Larval Size

**Figure 3:**
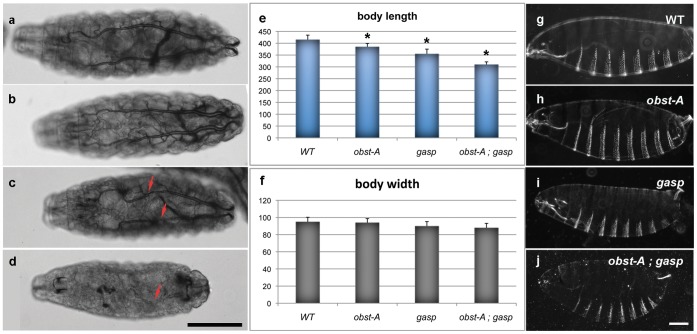
Gas filling and cuticle defects in *obst-A; gasp* mutant. (a–d) Bright field photomicrographs of wild-type (a), *obst-A* (b), *gasp* (c) and *obst-A; gasp* (d) first instar larvae. Refracted light makes the gas filled trachea clearly visible in the wild-type and mutant larvae. The trachea is not gas filled in the double mutant. Arrows indicate collapsed regions in the tubes. Scale bar: 100 µm (e, f) Body size measurement of wild-type, *obst-A*, *gasp* and *obst-A; gasp* first instar larvae. Body length (e) and body width (f) (n = 10). * Indicates the statistical significance (*p*<0.0001) of the difference between wild-type and each mutant as estimated by a two-tailed distribution unpaired Student’s *t*-test. (g–j) Dark field of cuticle preparations of wild-type (g), *obst-A* (h), *gasp* (i) and *obst-A; gasp* (j) mutant embryos. *obst-A; gasp* double mutant shows dilated cuticle. Scale bar: 25 µm.

Obst-A and other proteins involved in the assembly of chitinous extracellular matrices are essential for larval growth and cuticle integrity [28]. We first examined the anticipated role of Gasp in larval size control and cuticle formation. We analyzed the size and cuticle structure of *obst-A* and *gasp* mutants at the first instar larval stage (Fig. 3). Both *obst-A* and *gasp* single mutant larvae are shorter compared to heterozygote siblings. The body length of *obst-A* larvae was reduced by 8% and the length of *gasp* mutants was 14% shorter compared to the wild-type. Given the similarities of the encoded proteins and the resemblance of the expression patterns of the two genes we generated and analyzed *obstA; gasp* double mutants. *obstA; gasp* larvae were even shorter than *obstA* or *gasp* single mutants. Their body length was reduced by 25% compared to the wild-type larval length (Fig. 3a–e). The width of the mutants was not significantly different than the width of wild-type larvae (Fig. 3f). The increased severity of the body length defect in double mutants suggests that Obst-A and Gasp have overlapping additive roles in regulating larval length. We compared cuticle preparations of first instar larvae of the three mutant genotypes to wild-type preparations to assess the potential roles of role of *obst-A* and *gasp* in epidermal differentiation (Fig. 3g–j). We did not detect any obvious patterning defects or dorsal closure and head involution phenotypes in the single mutants or in *obst-A; gasp* animals. However, in contrast to the wild-type and single mutant preparations the cuticles of *obst-A; gasp* double mutants were severely bloated. This cuticle defect in the double mutants suggests a synergistic function for Obst-A and Gasp in chitin fibril assembly and exoskeleton integrity, which subsequently defines the shape and growth of the larva.

### Obst-A and Gasp are Required for Apical Matrix Integrity in the Airways

The remarkable intensification of the cuticle shape defect in the double mutants prompted us to examine the phenotypes of single and *obst-A; gasp* double mutants in the tracheal airways. A distinguishing feature of a functional tracheal respiratory network is the presence of gas in the larval tubes. Similar to wild-type controls, the airways of single *obst-A* and *gasp* mutant embryos became gas-filled at the end of embryogenesis (Fig. 3a–c). However, double mutant embryos showed a fully penetrant gas-filling defect (Fig. 3d). Additionally, *gasp* mutants showed regions of local tube collapse (Fig 3c), which could be rescued by re-expression of Gasp in the trachea of 89% of the mutants using the *btl*Gal4 driver (n = 28). A similar tube collapse phenotype was detected in the tubes of *obst-A; gasp* mutants (Fig 3d). This phenotype is common among mutants with defective epithelial integrity. Therefore, we analyzed the epithelial apical cell membrane and adherens (AJ) and septate junction (SJ) integrity by staining single and double mutants for Crumbs, *D*E-cadherin and MTf [23]. Neither the single nor the *obst-A; gasp* mutants show notable defects in the intensity or localization of these markers suggesting that epithelial integrity is not affected in the double mutants (Fig. S2). To identify the origin of the tube collapse phenotype in *obst-A; gasp* embryos we analyzed the luminal chitin filament and the apical taenidial lining of the tubes.

**Figure 4:**
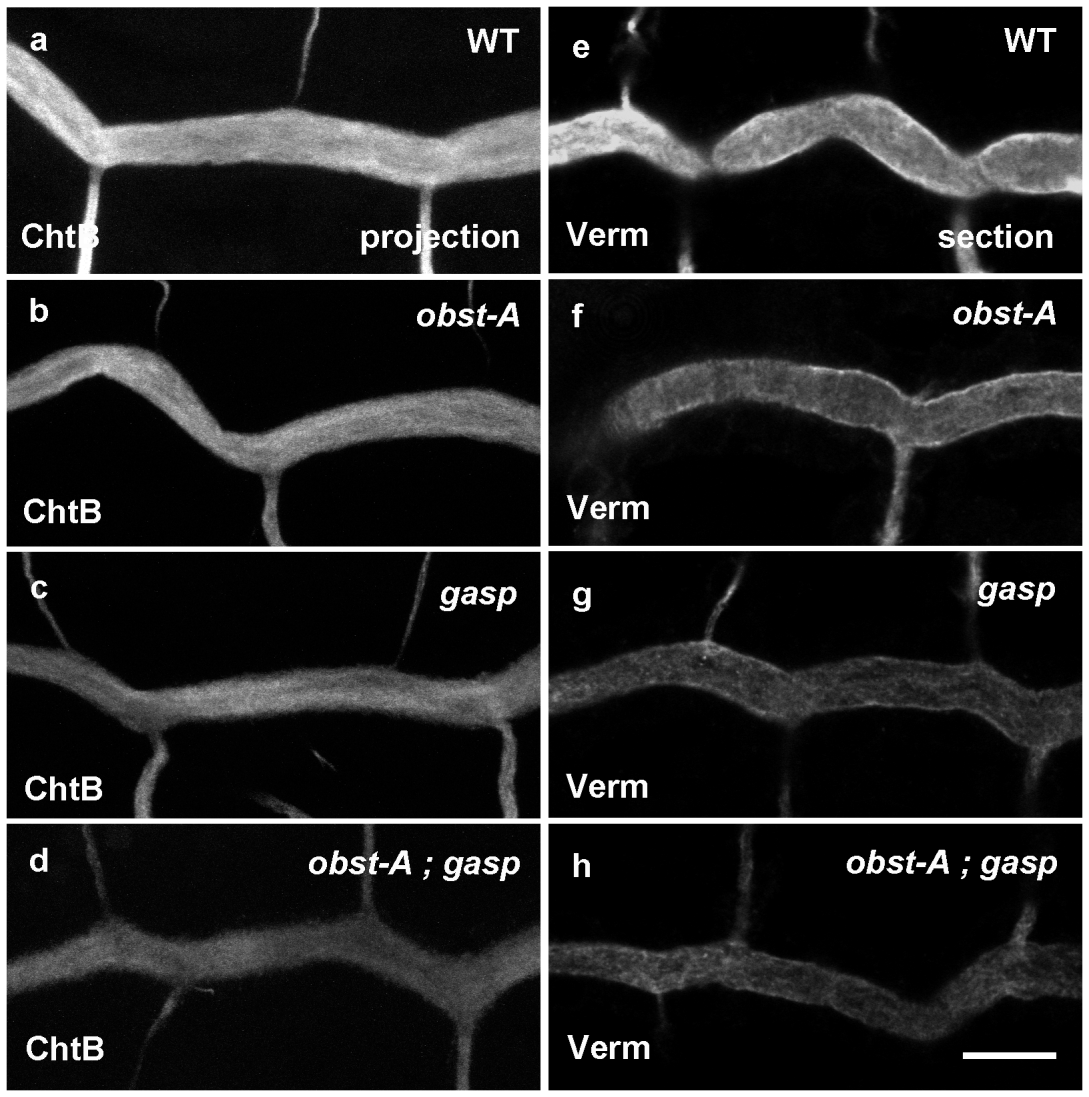
*obst-A* and *gasp* mutants show defects in luminal tracheal matrix. (a–d) Confocal microscopy projections of the trachea labeled with a FITC-conjugated chitin-binding probe (ChtB). The filamentous chitin is detected in the wild-type embryos at stage 16 (a). The chitin staining intensity is weaker in the *obst-A* (b), *gasp* (c) and *obst-A; gasp* (d) mutant embryos. (e–h) Optical sections of tracheal tubes stained for Verm. Verm is secreted to the tracheal lumen in wild-type embryos at stage 16 (e). The amount of secreted Verm is reduced in both *obst-A* (f) and *gasp* (g) single mutant embryos and *obst-A; gasp* (h) double mutant embryos. Scale bar: 10 µm.

Chitin is detected inside the dorsal trunk tubes of wild-type embryos at stage 14. It forms a cable of fibrils, which expands in diameter concurrently with the radial growth of the lumen before it disappears late at stage 16 [9]. We examined the transient luminal chitin cable and its deposition by using a fluorescent chitin-binding probe (ChtB) (Fig. 4). Both *obst-A* and *gasp* single mutants showed a decreased and irregular staining intensity of luminal chitin compared to wild-type embryos (Fig. 4b, c). In *obst-A;gasp* double mutants the staining of the intraluminal matrix was even more diffuse compared to the single mutants and to wild-type embryos (Fig. 4d). This suggested that the putative chitin binding proteins Obst-A and Gasp control the assembly or stability of the extracellular matrix. We used an antiserum against the secreted chitin deacetylase, Verm to examine if the extracellular matrix defect may be due to aberrant modifications of the chitin polysaccharides [13]. Luminal Verm staining was reduced in both *obst-A* and *gasp* single mutants and was further diminished in the double mutant (Fig. 4e–h). Importantly, the transcript levels of Verm were not affected in *obst-A* or *gasp* mutants compared to wild-type embryos indicating that Gasp and Verm control the luminal levels of Verm post-transcriptionally (Fig. S3).

**Figure 5:**
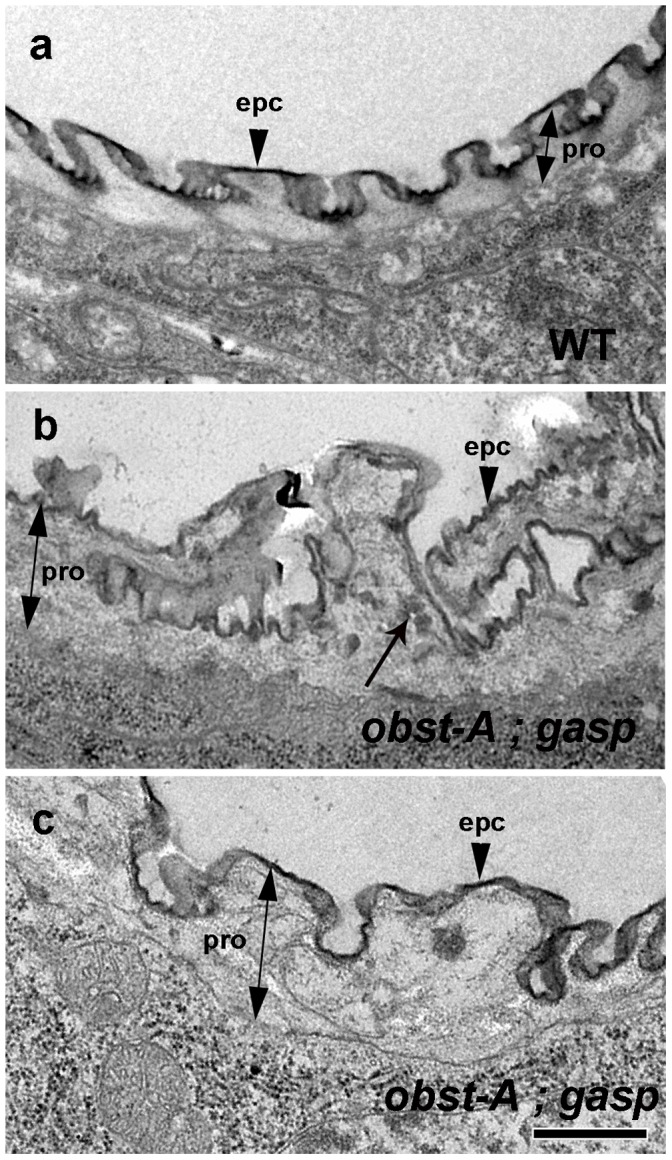
Ultrastructure of the tracheal lumen in *obst-A; gasp* mutants. (a–c) Transmission electron micrographs of wild-type and *obst-A; gasp* dorsal trunks at the end of embryogenesis. The wild-type embryos (a) show uniformly distributed chitin rich procuticle. In *obst-A; gasp* double mutant (b, c) the taenidial folds are irregular and the procuticle is distorted with granular amorphous accumulations (arrow). epc = epicuticle (arrow heads), pro = procuticle layer (double arrows). Scale bar: 0.5 µm.

To further test the potential structural role of Obst-A and Gasp in chitin assembly, we analyzed the chitin in the taenidia, the cuticular ridges that line and reinforce the airway tubes (Fig. 5). Transmission electron microscopy (TEM) reveals three layers of cuticle in wild-type embryos: the envelope layer, the proteinaceous epicuticle and the chitin-rich procuticle (Fig. 5a). TEM analysis of *obst-A; gasp* double mutants revealed a grossly irregular shape in the taenidial folds (Fig. 5b, c). The outermost layers of the envelope, and the dark epicuticle appeared normal in the mutants. However, the chitin-rich space between the epicuticle and the apical epidermal surface was disorganized and granular with amorphous composition compared to the uniform procuticle layer of the wild-type embryos.

Thus, the reduced staining intensity of the luminal chitin cable and the defects in the procuticle layer and the irregular taenidial folds in *obst-A; gasp* double mutant embryos suggest a direct function of Obst-A and Gasp in chitin fibril organization and maintenance.

### Obst-A and Gasp Drive Diametric Tube Expansion

**Figure 6:**
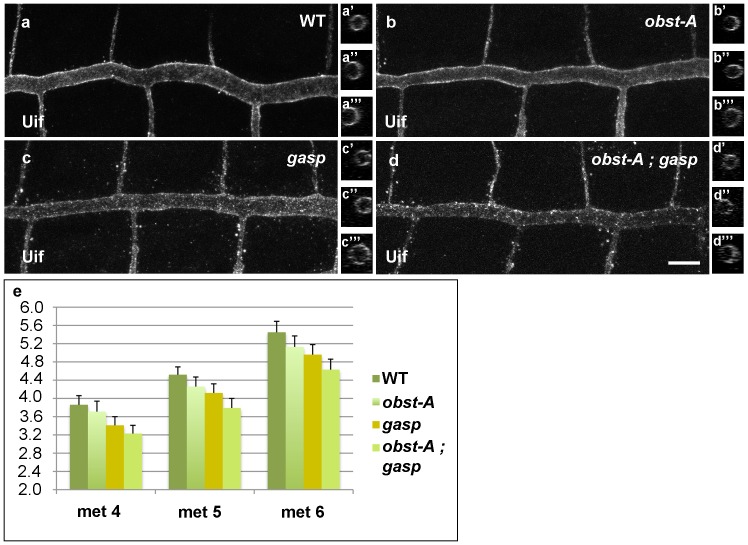
Lumen diameter expansion requires Obst-A and Gasp. (a–d) Confocal microscopy projections of the trachea of wild-type (a–a′′′), *obst-A* (b–b′′′), *gasp* (c–c′′′) and *obst-A; gasp* (d–d′′′) embryos labeled with the apical marker Uninflated (Uif) at stage 16.1. Inserts display y-z projections of the DT tubes from 3 different metameres. a′, b′, c′ and d′ inserts are from the middle of metamere 4, a′′, b′′, c′′ and d′′ from the middle of metamere 5 and a′′′, b′′′, c′′′ and d′′′ from the middle of metamere 6. Scale bar: 10 µm (e) Quantification of tracheal diameter of wild-type, *obst-A*, *gasp* and *obst-A; gasp* embryos at stage 16.1. The graph shows diameter measurements of three metameres: 4, 5 and 6. Number of embryos used for measurement of each genotype n = 6. The y-axis represents the diameter in micrometers. Tube diameter values in all mutant embryos were significantly different (p<0.05) from the wild-type by a two-tailed distribution unpaired Student’s *t*-test.

A characteristic phenotype in many mutants with disrupted apical extracellular matrix is the presence of overelongated or cystic tubes. To assess the impact of Obst-A and Gasp function in tube shapes and size control we assessed tracheal tube dimensions in single and double mutant embryos at stage 16. We stained *obst-A* and *gasp* single mutants for the apical membrane markers Crumbs and Uninflatable (Uif) [29] (Fig. 6a–d). Because the airway tubes are tapered we evaluated dorsal trunk (DT) length and diameter in three central consecutive tracheal metameres to obtain a representative view (n = 6). *obst-A* and *gasp* single mutants showed a small but significant reduction in airway diameter. In *obst-A* mutants we found an average 5% reduction in tube diameter, whereas in *gasp* mutants DT diameter was reduced by 10% compared to wild-type embryos. In *obst-A; gasp* double mutants the airway diameter was decreased by 15% in comparison to wild-type (Fig. 6e). By contrast we did not detect any significant changes in the length of the DT *obst-A; gasp* double mutants at stage 16 (Fig. S4). However, a portion of the *obst-A* and *obst-A; gasp* embryos also showed some tube tortuosity at stage 17, presumably due to the reduction in the luminal levels of Verp or Serp. This suggests that the primary role of the luminal proteins Gasp and Obst-A is to facilitate radial tube expansion. This function likely involves their role in the assembly of the chitin cable or the taenidial lining, which could provide a distending scaffold force that stretches the apical surfaces of the epithelial cells.

**Figure 7:**
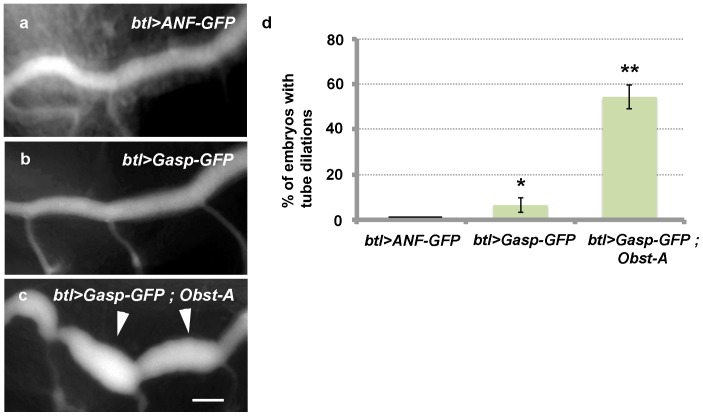
Overexpression of *Obst*-family members causes tube dilation. (a–c) Overexpression in wild-type embryos of *btl*>ANF-GFP (a), *btl*>*Gasp*-GFP (b) and *btl*>*Gasp*-GFP; *Obst-A* (c). The simultaneous overexpression of Gasp-GFP and Obst-A in trachea forms tube dilations in dorsal trunk (arrowheads) (c). (d) Quantifications showing the percentage (%) of embryos with dorsal trunk dilations in the following genotypes: *btl*>ANF-GFP (n = 16), *btl*>*Gasp*-GFP (n = 12) and *btl*>*Gasp*-GFP; *Obst-A* (n = 38). Bars show the means of three independent experiments and error bars show the ± standard error of the means. * and ** denote significant differences (p<0.01 and p<0.0001) between the means of *btl*>*Gasp*-GFP or *btl*>*Gasp*-GFP;*Obst-A* to the *btl*>ANF-GFP (control) by a two-tailed distribution unpaired Student’s *t*-test. Scale bar:10 µm.

To further test this hypothesis, we overexpressed Gasp-GFP alone or Gasp-GFP together with Obst-A or an unrelated secreted protein ANF-GFP in the trachea of wild-type embryos and assessed their ability to induce tube dilations (Fig. 7a–c). We monitored tube shape changes by live imaging, taking advantage of Gasp-GFP or ANF-GFP, which labeled the tracheal lumen. Obst-A, Gasp-GFP double overexpression caused local dilations in the DT tubes in 58% of the analyzed embryos (Fig. 7d). In these embryos the tubes were only partly cleared from luminal protein and collapsed during the subsequent step of liquid clearance (Movie S2). Gasp-GFP expression alone had only a minor effect in tube dilation and overexpression of the exogenous luminal marker ANF-GFP did not cause any phenotypes (Movie S1 and Fig. 7a). These results suggest that a concurrent increase in the luminal levels of both Obst-A and Gasp in the tracheal tubes can directly stimulate tube diameter expansion, presumably by modifying the structure of the luminal chitin. The subsequent tube collapse defect indicates that the excess of luminal chitin binding proteins interferes with the biophysical properties of the matrix and leads to airway collapse during the process of liquid clearance.

## Discussion

We have identified Gasp as the unknown antigen of the 2A12 mAb, which has been the major luminal marker for the analysis of tracheal airways in the last 20 years. The 2A12 antigen together with the apical protein Piopio represent a group of endogenous secreted cargoes that selectively rely on the formin protein, Diaphanous for their secretion [30]. On the other hand, Verm and Serp exemplify luminal proteins that require alternative targeting routes involving the septate junctions and endosomal compartments for their luminal accumulation [13], [31]. The identification of the 2A12 antigen further enables the investigation of the targeting signals determining the luminal deposition of tube size regulators (Gasp) and other apical cargos.

Our genetic analysis and the predicted protein sequences strongly argue that both Obst-A and Gasp are required for chitin extracellular matrix assembly and integrity. Both the chitin staining and the levels of the putative chitin deacetylase Verm are reduced in *obst-A* and *gasp* mutants. This is in accord with biochemical and histological analysis of Obst-A, which has been shown to bind to chitin and other chitin binding proteins and modifying enzymes [28].

We showed that the Gasp and Obst-A duo present the first apical ECM proteins directly involved in diametric tube expansion in the *Drosophila* airways. Mutants lacking both proteins show a significant decrease of 15% in DT diameter compared to wild-type. Zygotic null *sar1* mutants show a 30% reduction in tube diameter and a severe defect in ER integrity and the apical secretion of all ECM proteins tested [17]. This comparison suggests that Obst-A and Gasp account for a substantial subset of the secreted proteins that directly drive diametric expansion. This is further supported by the tube dilation defects generated by the concurrent overexpression of both proteins in the tracheal epithelium. However, neither the overexpression of Obst-A nor Gasp alone could generate a notable diametric increase in the tubes of wild-type embryos. This suggests that the luminal amount of the two proteins need to reach a critically high level to drive tube expansion. Alternatively, and despite the similarities in amino acid sequence and domain organization, either of the two proteins may influence chitin assembly differently and they may both be needed to achieve the optimal structural modifications. Petkau et al. noticed a late defect of tube overelongation in *obst-A* mutants. We noted that this phenotype appears at later stages compared to the diametric expansion phenotype of *obst-A* and *obst-A; gasp* mutants. We therefore argue that the tube overelongation defect is a secondary function of the tracheal Obst family members and is likely attributed to the reduction of the luminal levels of Verm and Serp.

How does chitin modification in the lumen lead to diametric tube expansion? Our data suggest that Obst and Gasp organize luminal chitin fibrils into an expanding structure that generates an intra-luminal mechanical tension forcing the apical surface of the underlying epithelial cells to expand. This dilation mode depending on local assembly and swelling of the luminal matrix also operates in the hindgut, where instead of chitin it involves mucin-type glycoproteins [32]. In the tracheal tubes, the secretion of chitin-binding proteins and modifying enzymes would lead to their direct association with the luminal chitin in the vicinity of their secretion. Thereby, secreted tube size regulators, like Obst-A and Gasp would exert the local dilation effects observed around secretion-defective *sec24* mutant cells overexpressing sec24 [19].

The Obstructor gene family is conserved across invertebrate species ranging from nematodes to arthropods and the family members are expressed in variety of tubular organs and the epidermis [26]. Our genetic analysis of *obst-A* and *gasp* suggests central roles of *obst* genes in the morphogenesis of the majority of the identified animal species. Additionally, the analysis of of *obst-A* and *gasp* mutants argues for the important role of apical extracellular matrix assembly in tube dilation. Glycan-rich apical matrices line the surface of several tubular organs in vertebrates and may similarly participate in shaping the final tube dimensions during their morphogenesis [33]–[35].

## Supporting Information

Figure S1 **Characterization of the** ***obst-A*** **and** ***gasp*** **mutants.** (a, b) Whole-mount in situ hybridization of *obst-A* heterozygous (a) and *obst-A* mutant (b) embryos with *obst-A* probe and GFP antibody. GFP staining discriminated heterozygous embryos carrying the balancer chromosome. The *obst-A* mRNA is not detected in *obst-A* mutant embryos. (c, d) Wild-type (c) and *gasp* mutant (d) embryos stained with antibodies against the Gasp protein. Gasp staining is not detected in *gasp* mutant embryos. Scale bar: 25 µm.
(TIF)

Figure S2 **Apical membrane, AJs and SJs are not affected in the** ***obst-A*** **and** ***gasp*** **mutants.** Confocal microscopy sections of the tracheal dorsal trunk labeled with the apical marker Crumbs (Crb) (a–d), the adherens junctions marker *D*E-cad (e–h) and the septate junction marker MTf (i–l). Crb is localized apically both in wild-type (a) and *obst-A* (b), *gasp* (c), *obst-A; gasp* (d) mutant embryos. *D*E-cad and MTf localization is the same in wild-type (e) and *obst-A* (f), *gasp* (g), *obst-A; gasp* (h) mutant embryos. (m, n) Confocal microscopy projections of the wild-type (m) and *obst-A; gasp* (n) mutant trachea, labeled with *D*E-cad. Scale bar: 10 µm.
(TIF)

Figure S3 **Transcript levels of Verm are not changed in** ***obst-A*** **or** ***gasp*** **mutants.** (a–d) Whole-mount in situ hybridization of wild-type (a), *obst-A* (b), *gasp* (c) and *obst-A; gasp* (d) embryos with *verm* probe. Scale bar: 25 µm (e).
(TIF)

Figure S4 **The DT length is not affected in** ***obst-A*** **or** ***gasp*** **mutant embryos.** (a) Quantification of tracheal length of wild-type, *obst-A*, *gasp* and *obst-A; gasp* embryos at stage 16.1. The graph shows length measurements of three metameres: 4, 5 and 6. Number of embryos used for measurement of each genotype n = 6. The y-axis represents the length in micrometers. No significant difference was detected (p>0.05) in comparison between wild-type embryos with different mutants (two-tailed distribution unpaired Student’s *t*-test).
(TIF)

Movie S1 **Wide-field, time-lapse movie showing the DT of a wild-type embryo expressing** ***btl*** **>** ***Gasp*** **-GFP at late stage 16.** Images were acquired every 10 min and for a period of 90 min.
(MOV)

Movie S2 **Wide-field time-lapse movie showing the DT of a wild-type embryo expressing** ***btl*** **>** ***Gasp*** **-GFP;** ***Obst-A*** **at late stage 16.** The trachea develops tube dilations in the dorsal trunk and finally collapses. Images were acquired every 10 min and for a period of 90 min.
(MOV)
