# Supplementary figures and images for: Control of Airway Tube Diameter and Integrity by Secreted Chitin-Binding Proteins in Drosophila
Source: PLoS One. 2013 Jun 24;8(6):e67415. doi: 10.1371/journal.pone.0067415 (PMC3691276; doi:10.1371/journal.pone.0067415)

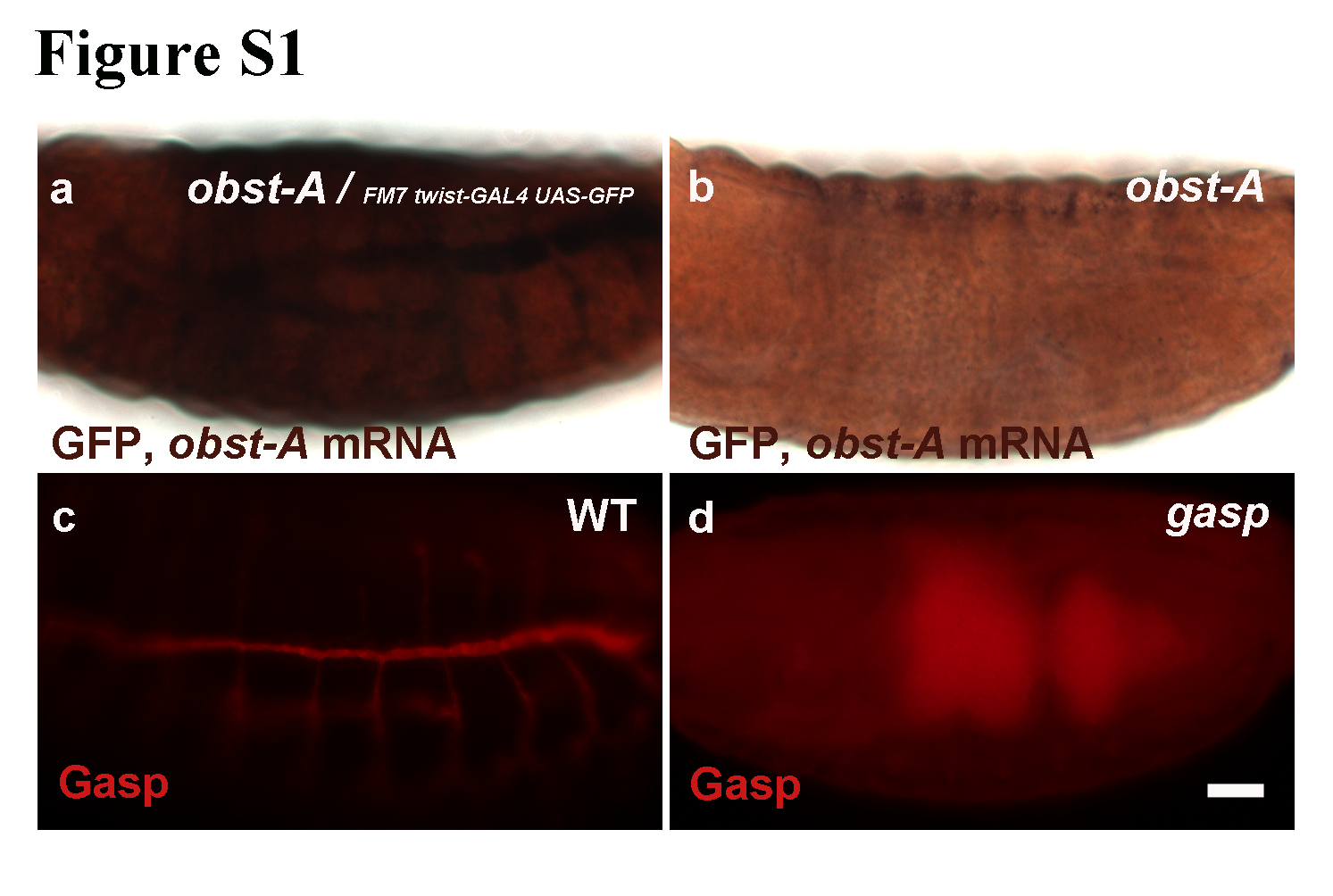

Supplement: Figure S1 — Characterization of the obst-A and gasp mutants. (a, b) Whole-mount in situ hybridization of obst-A heterozygous (a) and obst-A mutant (b) embryos with obst-A probe and GFP antibody. GFP staining discriminated heterozygous embryos carrying the balancer chromosome. The obst-A mRNA is not detected in obst-A mutant embryos. (c, d) Wild-type (c) and gasp mutant (d) embryos stained with antibodies against the Gasp protein. Gasp staining is not detected in gasp mutant embryos. Scale bar: 25 µm. (TIF) [file pone.0067415.s001.tif]

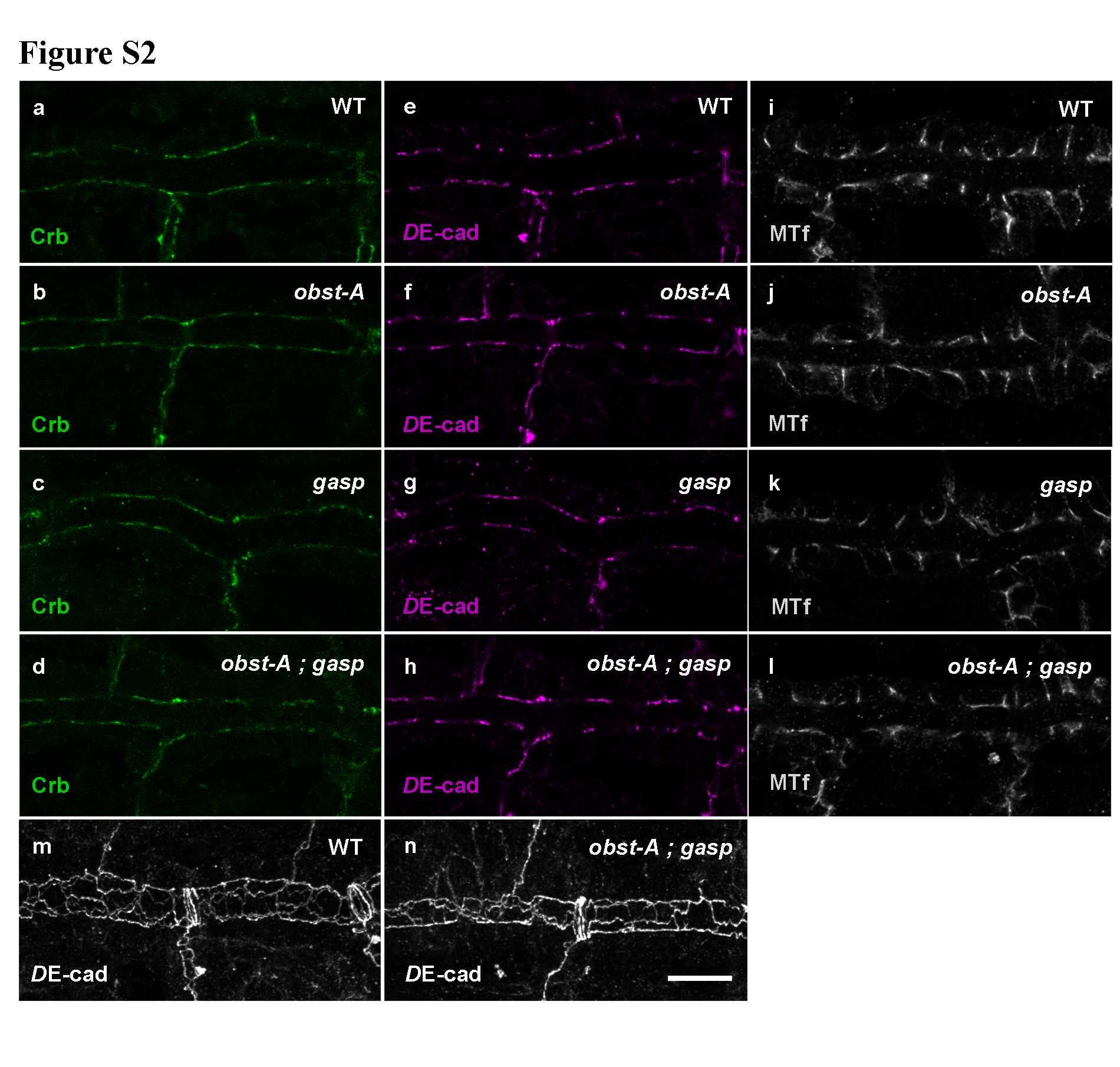

Supplement: Figure S2 — Apical membrane, AJs and SJs are not affected in the obst-A and gasp mutants. Confocal microscopy sections of the tracheal dorsal trunk labeled with the apical marker Crumbs (Crb) (a–d), the adherens junctions marker DE-cad (e–h) and the septate junction marker MTf (i–l). Crb is localized apically both in wild-type (a) and obst-A (b), gasp (c), obst-A; gasp (d) mutant embryos. DE-cad and MTf localization is the same in wild-type (e) and obst-A (f), gasp (g), obst-A; gasp (h) mutant embryos. (m, n) Confocal microscopy projections of the wild-type (m) and obst-A; gasp (n) mutant trachea, labeled with DE-cad. Scale bar: 10 µm. (TIF) [file pone.0067415.s002.tif]

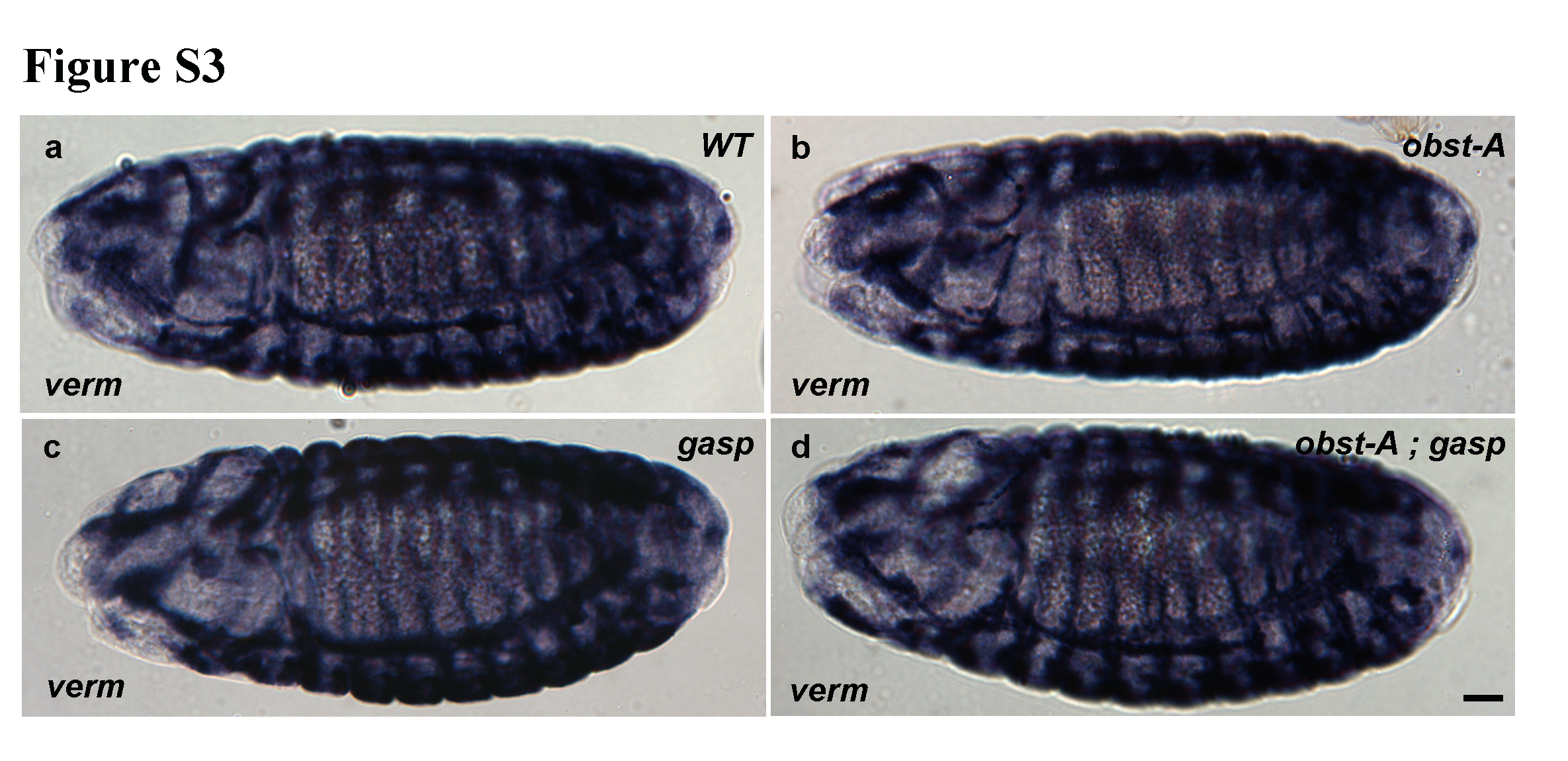

Supplement: Figure S3 — Transcript levels of Verm are not changed in obst-A or gasp mutants. (a–d) Whole-mount in situ hybridization of wild-type (a), obst-A (b), gasp (c) and obst-A; gasp (d) embryos with verm probe. Scale bar: 25 µm (e). (TIF) [file pone.0067415.s003.tif]

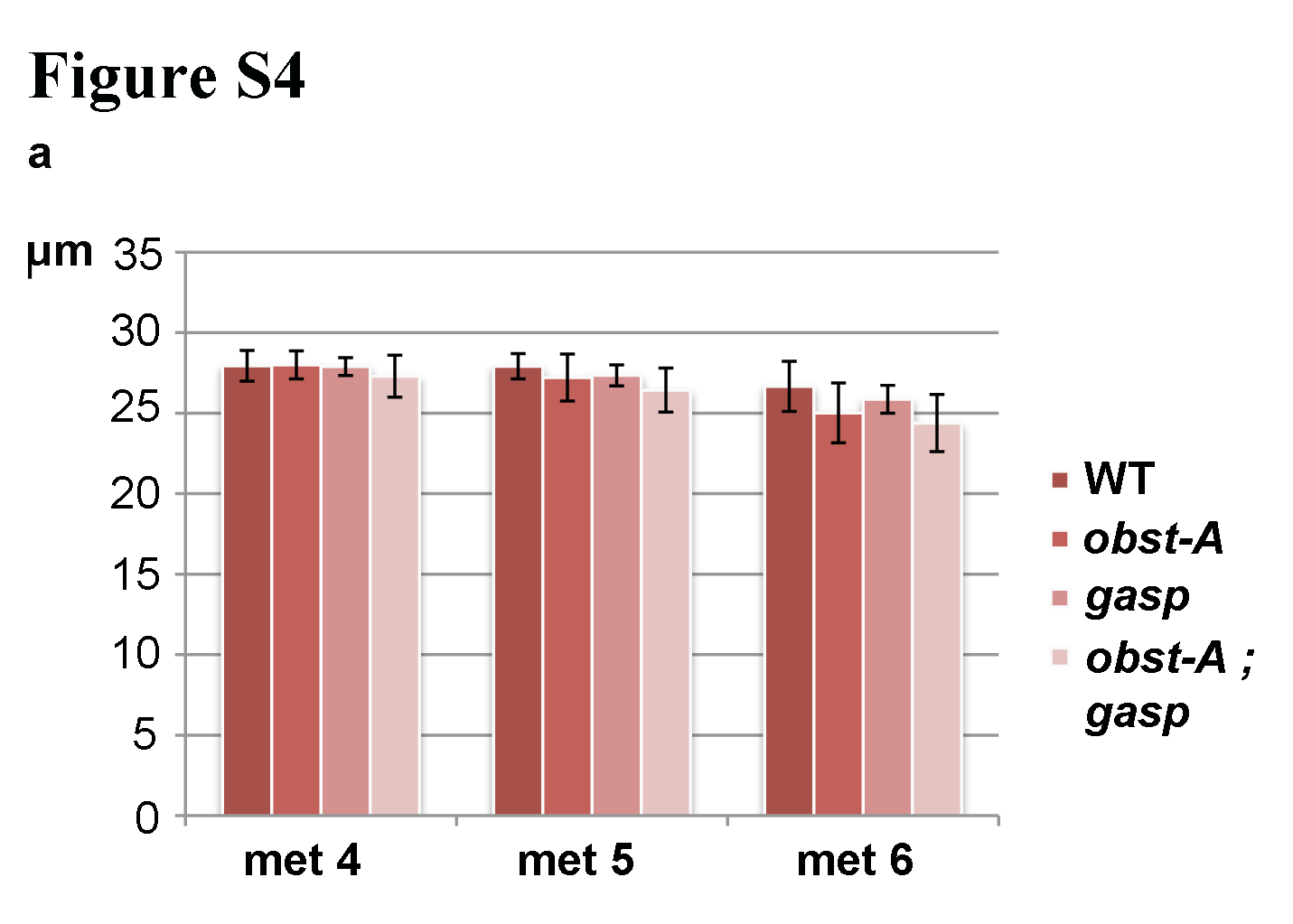

Supplement: Figure S4 — The DT length is not affected in obst-A or gasp mutant embryos. (a) Quantification of tracheal length of wild-type, obst-A, gasp and obst-A; gasp embryos at stage 16.1. The graph shows length measurements of three metameres: 4, 5 and 6. Number of embryos used for measurement of each genotype n = 6. The y-axis represents the length in micrometers. No significant difference was detected (p>0.05) in comparison between wild-type embryos with different mutants (two-tailed distribution unpaired Student’s t-test). (TIF) [file pone.0067415.s004.tif]
